# Death Anxiety in Social Workers as a Consequence of the COVID-19 Pandemic

**DOI:** 10.3390/bs11050061

**Published:** 2021-04-26

**Authors:** José Ángel Martínez-López, Cristina Lázaro-Pérez, José Gómez-Galán

**Affiliations:** 1 Department of Social Work and Social Services, University of Murcia, Avda. Teniente Flomesta, 5, 30003 Murcia, Spain; jaml@um.es; 2 Department of Political Sciences, Social Anthropology and Public Finances, University of Murcia, C/Campus Universitario, 11, 30100 Murcia, Spain; cristina.lazaro2@um.es; 3 Department of Education, University of Extremadura, Avda. de Elvas, s/n, 06006 Badajoz, Spain; 4 Cupey Campus, Ana G. Méndez University, San Juan, PR 00926, USA

**Keywords:** social workers, death anxiety, health crisis, prevention, COVID-19

## Abstract

The COVID-19 pandemic has affected all social spaces, conditioning our daily routines, including those at work. All professions have been affected by stressful situations and anxiety in the proximity’s face of death generated by the pandemic. In this context, some professionals have emerged as essential, as social workers, acting in extreme situations in the face of increased demands and social uncertainty arising from the health crisis. The present study aimed to determine the levels of anxiety about death among social workers in Spain. For this purpose, an ad hoc questionnaire was designed, taking the Collett and Lester Fear of Death Scale as a reference (*n* = 304). The exploitation of the data was carried out from a quantitative perspective. First, a descriptive analysis was performed. Then, binary logistic regressions were carried out on the general scale. The dependent variable in all of them was the risk of suffering death anxiety to the set of its subscales. The main research results show high values of this anxiety in social workers concerning the general value of the scale—and the subscales—and the point of view of state and process. The highest values were Fear of Death of Others (81.6%) and Fear of the Process of Dying of Others (78.3%). Regarding the binary logistic regressions applied, predictor variables were identified in all of them, but the following stand out: Lack of personal protection equipment and Need psychological or psychiatric support. In addition, being a woman increases the risk of suffering Fear of the Dying Process of others.

## 1. Introduction

The pandemic that has hit the entire planet since the SARS-CoV-2 coronavirus became known in December 2019 has shown that many professions today are considered essential, and more so than ever. In Spain, since the State of Alarm decree [1] was established in March 2020, it has been assumed that professions, such as healthcare, security forces, and corps, transporters, gas station workers, airports, or media, have had an essential role in the supply and provision not only of physical means for subsistence during the months of confinement and beyond but also in the care and protection professions.

This situation, in which the spread of the virus occurred worldwide, revealed the many personal and social needs and shortcomings that, although existing, had not been as visible as in the post-confinement period. This context raises the question of the extent to which institutions are prepared to professionally respond to emerging situations in public health and the social field. In this sense, social workers’ work is becoming essential to ensure the welfare, especially of the most vulnerable and fragile people, lacking the most basic needs. This is the case of people living on the street, the elderly, people with disabilities, or those in need of long-term care, among others. Social work is, therefore, an essential profession in the field of social policy [2,3].

The worrying situation from which 2021 society has not yet recovered has generated a significant workload in this professional group working in the social field. Its main mission is to act in inclusive projects, promoting equality, autonomy [4], social justice [5], and to expand and maintain social protection to ensure the welfare state in terms of health, guaranteed income and pensions, education, and social services, even innovating new models of social care in times of great social difficulty [6,7].

The pandemic has put public social services under intense stress and has generated a great inequality and social gap that social work tries to minimize. Different publications [8,9,10] have echoed this situation, stating as the main factor the impact of the cessation of economic activity, which has led to a minimization of family income and aggravated inequality if different social groups are compared, affecting education, housing, food, or health. That is why it would be a challenge for society to ensure social coverage to minimize this high social gap, being social workers the professionals in charge of providing and/or managing social benefits and interventions that result in the welfare improvement.

## 2. Background

Several studies have been carried out on individuals’ mental health because of their association with certain professions, especially those related to the health field [11,12,13,14]. Although social sciences, specifically with social workers, this issue has been less studied, we can find some relevant studies [15,16,17,18].

The current health crisis highlights the need for professionals who work directly with people to be prepared to face extreme conditions, not only stressful due to the urgency of the emerging situation but also because of the anxiety generated by working in emotional exhaustion situations a pandemic. Here, and considering aspects, such as the number of deaths in Spain due to the SARS-CoV-2 coronavirus, at 83,706 according to the National Institute of Statistics (*Instituto Nacional de Estadística*) [19], the situation of marginalization in which new families find themselves, besides the social problem existing in Spain, are sufficient factors to cause emotional reactions by social workers that require professional psychological intervention.

Some studies have been found concerning death anxiety of social workers in palliative care because they are subjected to an environment of high stress and many losses [18,20]. With the COVID-19 pandemic, human, material, social, etc., losses have been increased for many months in a row, and social workers have had to deal with the personal pain that losing users can cause them while feeling their grief and that of their families. These experiences can trigger emotional stress and anxiety in the face of the social worker’s death [21].

However, many of the demands of social workers during the first wave of the pandemic were more about the lack of certain services and resources to adequately develop their work activity in the telework format [22,23,24] than about the coping of the situation of the people with whom they worked, within a context of increased anxiety as a consequence of the confinement of the population. Some studies have analyzed the effects of the fear and anxiety caused by the pandemic in various groups [25,26], maximized by the impact generated by the media [27].

For all these reasons, it is essential to analyze death anxiety in essential professionals in the current social and health care field due to the implications it can have at the individual, social and occupational levels. High levels of death anxiety can cause mental health problems, including permanent anxiety and depression, among other pathologies, favoring these professionals’ greater vulnerability to extreme situations, such as those experienced during the exercise of their work [28,29,30].

Researchers’ situation generated by the pandemic has led to great interest in knowing the mental state of many workers considered essential during this stage. The chances of developing disorders, such as post-traumatic stress, anxiety, or depression, are very high in this type of overwhelming situation [12,31,32]. With health workers and security forces, because of their high exposure to the virus and the problem of heightened physical and emotional stress, high levels of death anxiety were found [11,33].

Social workers have also been living in an alarming situation that may develop emotional distress without adequate psychological resources [34] and anxiety. They have been in contact with the reality of many individuals and families who in the last year have seen not only their health but also their income endangered, thus generating a greater need for fundamental issues. For this reason, the present research is pertinent to know how the pandemic has affected this group of workers in the field of death anxiety.

The origin of the emotion that gives rise to fear and, consequently, death anxiety is diffuse and can be established in different causes. Thus, it is more complicated to set a concrete form of manifestation. Collett and Lester [35] developed a Fear of Death scale, marking the multiple causes that can cause it. Different publications [36,37] emphasized that the manifest reactions will depend on anxiety as a state or a trait. Collett and Lester distinguished four components: fear of one’s death, fear of the death of others, fear of one’s dying process, and fear of the dying process of others [29,38].

## 3. Materials and Methods

### 3.1. Objectives

The aim of the present study is twofold. On the one hand, to determine the levels of death anxiety in Spain’s social workers during the health crisis derived from COVID-19 (OG1). Second, taking the Collett and Lester death anxiety scale [35] as a reference, whether there are differences between its different components (OG2).

The hypothesis guiding the research is that social workers, being one of the essential groups that have worked close to the COVID-19 context, will show high values of death anxiety, especially about the fear of the death of others and the fear of the dying process of others.

### 3.2. Measures

In the approach to the object of study, the Collett-Lester Fear of Death Scale [35], validated by Venegas et al. [39] was used, in which reliability coefficient (Cronbach’s alpha) was 0.91 for the subscale fear of one’s death, 0.92 for fear of one’s dying process; fear of the death of others obtained 0.88 and 0.92 for fear of the dying process of others [40,41]. This scale is widely used in the health care setting. Still, it can apply to any professional group, especially where there has been proximity to death, as of social workers during the current pandemic. This scale comprises four blocks: “Fear of one’s Death” (AM1), “Fear of one’s own Dying Process” (AM2), “Fear of the Death of Others” (AM3), and “Fear of the Dying Process of Others” (AM4). Each of these components includes seven items in which response possibilities are structured through a Likert scale from 1 (not at all) to 5 (very much).

This variable was established as dichotomous: the existence of anxiety about death or not. The procedure for its determination was based on the average value got. If this was higher in the set of subscales, it shows anxiety about death from a general perspective of the scale. The same occurs with the values of the different components of the scale (four subscales). Thus, through this configuration of the dependent variable, following the objectives set, it is possible to get information from an internal and external perspective of the object of study and the subjects’ present situation as a process.

### 3.3. Variables Used

A. Dependent Variable. The dependent variable used is death anxiety in social workers, both in its general index and in the set of subscales.

B. Independent Variables. Two types of independent variables were established: (a) sociodemographic and (b) subjective perceptions of the current situation at work. The following were used regarding the sociodemographic variables: sex, age, and the area where the professional activity is carried out (Primary Care Social Services, Specialized Social Services, Health Social Services, Third Sector, and Other).

In Spain, there are significant differences between the professional fields of action of social workers. Primary Care Social Services are those provided by public administrations, but from a municipal perspective, i.e., closer to the citizen. Second, Specialized Social Services are those offered by a higher administrative entity than the municipal one and for specific groups, with specialized intervention and resources: the internment of minors in emergencies, management of benefits for the elderly or disabled, etc. Health Social Services are all those services that, from the health field, are provided to citizens from hospitals or health centers, but always from a public perspective, as in the two previous cases. The third Sector involves all the private or concerted entities that carry out social action actions. Finally, Other would include other minority social action sectors, such as penitentiary centers, courts, etc.

Concerning the independent variables related to subjective aspects linked to the development of their professional activity, the following were established: whether they need psychological or psychiatric support (NPPS); whether they believe that psychological or psychiatric support should be offered from work centers (PPSS); whether they feel they may need psychological or psychiatric support (PPSN); whether they feel that the lack of personal protection equipment increases their stress and anxiety levels (PPE); worked with COVID-19 during the first wave of the pandemic (WFW); whether they teleworked during the first wave of the pandemic (TL); whether they feel their work has been recognized by the organization they work for (ROW).

### 3.4. Procedure

The questionnaire was configured through a tool called *umu.encuestas*, dependent on the University of Murcia, and was distributed online, maintaining the subjects’ confidentiality. The field research was conducted from 1 September until 12 October 2020. Although it would have been interesting to implement the fieldwork earlier, difficulties in accessing participants delayed its implementation.

The approach to the object of study is quantitative through a simple random sampling in this professional group. The questionnaire was administered through the General Council of Social Work in Spain, which brings together the different territories’ professional associations. The study’s universe is unknown since no data was provided on how many members are part of this organization or how many could be sent the questionnaire. It was relied on this organization to get data from the entire Spanish territory when it was not possible to administer a questionnaire physically, and the workload and pressure at work because of the increase in social demands made it necessary to establish a methodological design that would allow reaching the participants quickly.

For the development of the research, the postulates of the Declaration of Helsinki were subscribed to, and all the participants gave their authorization to take part in the study. Likewise, all the Ethics Committees’ protocols of the universities to which the authors belong were followed. Although in Spain institutional approval is only required for experimental studies and not for descriptive studies, the Codes of Good Practice in Human Research were subscribed. The research was registered and signed with the number REPRIN-PEM-23 by the working group formed by the researchers who authored the study.

Regarding data exploitation, a descriptive analysis of the variables used was initially developed. Subsequently, a binary logistic regression was performed, taking as a reference the level of anxiety about death in general and each of the subscales. The data exploitation and analysis processes were carried out through the IBM SPSS V.24 program.

The variables introduced in the binary logistic regression analysis are shown in Table 1.

### 3.5. Participants

The total number of participants in this research was 304. A feature that is consistently observed in studies on social workers is their level of feminization in Spain, which in this case, reaches 88.3%. In terms of age, the largest population cohort is found among those between 41–50 years of age, which reaches 32.6%. Regarding the professional field, those who develop their professional activity in Primary Care Social Services stand out above all, as 37.1% of the total. Table 2 shows the rest of the sociodemographic characteristics.

## 4. Results

In a first approximation to the death anxiety scales results in social workers, it is observed that all of them show pro-average values above the mean. However, the scales related to Fear of the Death of Others (AM3) and Fear of the Dying Process of Others (AM4) stand out, with 81.6% and 78.3%, respectively. Although the general levels of death anxiety are high, they increase with others’ perception of death. The Total Death Anxiety scale (AM_T) stands at 68.8%. The lowest values are observed about Fear of Death itself (AM1) and Fear of the Dying Process itself (AM2), which stand at 57.6% and 59.9%, respectively.

Concerning NPPS, almost one in three people (29.2%) responded affirmatively to this question. However, about PPSN, 69.1% consider this need to be likely in the future. 86.8% consider that PPSS. These three results show a considerable difference between the present and future need for psychological treatment. This situation is framed within a work context where almost 9 out of 10 people consider that support should be provided to mitigate these problems from the work centers themselves.

Sixty-eight and a half percent of the participants consider that PPE. It should be regarded as that PPE, during the pandemic, marked the work dynamics of the residential centers, and, although not all WFW (48.0%), the stress and anxiety generated by this situation persist in the professionals. TL was a work activity followed by the social entities in most cases; 81.8% stated that they had worked from home. Finally, with the descriptive data, 79.9% ROW, a fundamental aspect to feel integrated or not within the labor structure and the institution’s purposes in which they work. The rest of the descriptive data can be seen in Table 3.

Concerning the multivariate analysis, five binary logistic regressions were applied, the dependent variable being the level of death anxiety both in its broad scale and in its four subscales. All the binary logistic regressions used showed adequate levels of fit.

AM1 presented a statistically significant model X2 = 19.721, *p* < 0.000. The model explains 8.9% (Nagelkerke’s R2) of high, moderate consumption variance and correctly classifies 61.7% of the cases. The Hosmer-Lemeshow test showed no significant difference between observed and predicted results in the model with a *p* = 0.928.

The variables included in the equation were: (a) Lack of personal protection equipment (PPE) and (b) subjective perception of needing psychological/psychiatric treatment because of COVID-19 (NPPS). Lack of personal protection equipment showed an odds ratio (OR) of 2.701 (95% CI, 1.548–4.714; *p* = 0.000). Regarding NPPS, it showed an OR of 1.929 (95% CI, 1.141–3.261; *p* = 0.014). Thus, PPE increased by almost three times (2.701) the probability to suffer AM1. Regarding the other predictor variable, NPPS, those who currently require psychological or psychiatric treatment are two times more likely (1.929) to suffer AM1.

AM2 presented a statistically significant model X2 = 11.202, *p* < 0.004. The model explained 5.2% (Nagelkerke’s R2) of high, moderate consumption variance and correctly classified 62.0% of the cases. The Hosmer-Lemeshow test showed no significant difference between the observed and predicted results in the model with a *p* = 0.963.

The variables included in the equation were the same as in AM1, although with slightly lower predictive values: (a) Lack of personal protection equipment (PPE) and (b) subjective perception of needing psychological/psychiatric treatment because of COVID-19 (NPPS). PPE showed an OR of 1.960 (95% CI, 1.171–3.281; *p* = 0.010). NPPS showed an OR of 1.754 (95% CI, 1.017–3.023; *p* = 0.043). Therefore, PPE influenced social workers so that those who felt anxiety or stress because of their absence were almost twice as likely (1.960) to suffer AM2. Similar results were observed in NPSS, where the subjective perception of needing this type of treatment made social workers up to 1.7 times more likely to suffer AM2.

AM3 presented a statistically significant model X2 = 22.058, *p* < 0.000. The model explains 11.6% (Nagelkerke’s R2) of high, moderate consumption variance and correctly classifies 81.9% of the cases. The Hosmer-Lemeshow test showed no significant difference between the observed and predicted results in the model with a *p* = 0.411.

The variables included in the equation coincide with AM1 and AM2 (Lack of personal protection equipment and Need psychological or psychiatric support). PPE showed an OR of 2.163 (95% CI, 1.145–4.088; *p* = 0.018). NPPS showed an OR of 2.863 (95% CI, 1.089–7.528; *p* = 0.023). Here, the variable with the greatest predictive power is NPPS, according to which, people who report currently feeling the need for treatment about their mental health see an increase of almost three times (2.863) in their chances of suffering anxiety about dying. PPE maintains similar values to the previous subscales, with participants showing up to 2 (2.163) times more possibilities of suffering AM3.

AM4 presents a statistically significant model X2 = 20.823, *p* < 0.000. The model explains 10.8% (Nagelkerke’s R2) of high, moderate consumption variance and correctly classifies 79.4% of the cases. The Hosmer-Lemeshow test showed no significant difference between the observed and predicted results in the model with a *p* = 0.874.

As in the previous predictive models, (a) Lack of personal protective equipment (PPE), and (b) Need psychological or psychiatric support (NPPS) were maintained as predictor variables, but a new variable was added: sex. PPE showed an OR of 2.398 (95% CI, 1.326–4.338; *p* = 0.004). NPPS showed an OR of 2.722 (95% CI, 1.290–5.743; *p* = 0.009). With sex, specifically being female, showed an OR of 2.425 (95% CI, 1.080–5.445; *p* = 0.032). In all cases, these variables predict the risk of suffering AM4 by almost 2.5 times. It is striking that being a woman is dying of others is a predictor variable compared to the rest of the subscales.

AM_Total presents a statistically significant model X2 = 19.005, *p* < 0.000. The model explains 9.0% (Nagelkerke’s R2) of high, moderate consumption variance and correctly classifies 67.9% of the cases. The Hosmer-Lemeshow test showed no significant difference between the observed and predicted results in the model with a *p* = 0.702.

The variables included in the equation were again: (a) Lack of personal protective equipment (PPE), and (b) Need psychological or psychiatric support (NPPS). PPE showed an OR of 2.348 (95% CI, 1.371–4.019; *p* = 0.002). NPPS showed an OR of 2.463 (95% CI, 1.320–4.596; *p* = 0.005). Thus, the odds of having Death Anxiety increased by 2.3 times if participants saw their anxiety/stress levels increase due to PPE and by 2.4 times if they felt they currently needed NPPS. The summary of the binary logistic regression models is presented in Table 4.

## 5. Discussion

The SARS-CoV-2 pandemic has prompted significant changes in how human beings deal with many of life’s realities. This study has revealed that social workers, in this context of the COVID-19 pandemic, have experienced a high level of fear of the dying process, both their own and others. In these circumstances in which the virus keeps sick people in need, most times assisted respiration, dying is more present than ever than death itself. In this sense, the high levels of fear of others’ dying process coincide with previous research concerning other groups, such as health care workers and law enforcement [11,42].

Both with the set of subscales of Death Anxiety and the group of subscales that compose it, high levels are observed in the participants, although those related to Fear of the Death of Others (81.6%) and Fear of the Process of Dying of Others (78.3%) stand out.

Another outstanding fact that evidence of anxiety and stress to which social workers are subjected is that almost one in three people have NPPS. However, if they are asked a time perspective whether they think they might need psychological or psychiatric treatment, these values rise to 69.1%. Therefore, although the present assessment of this situation is lower, partly due to the use of maladaptive coping strategies [43], the projection they make in the future is an indicative factor of these professionals’ emotional burden. Different investigations reach the same conclusions concerning the need for psychological help [44,45].

These levels are framed within the work environment. Perhaps this is one reason 86.8% consider that they should receive help from the work centers themselves and that almost 90% of the social workers do not feel represented by their institution. On the other hand, 68.5% of the participants consider PPE increased their stress or anxiety levels. It should be regarded as that PPE, during the pandemic, marked the work dynamics of the residential centers.

Significantly, although less than half of the respondents did not work directly during the first wave of the pandemic, and 81.8% of social workers teleworked, 68.5% perceived that their stress and anxiety levels increased because of the lack of PPE. In the essential professions, PPE has been a critical variable for developing a high emotional impact that has threatened workers’ mental health, as recent research has also pointed out [46,47]. Different studies have made visible the fear of many professionals who worked during the highest risk stages of contracting the virus because of the lack of adequate protection measures [48,49,50].

The results are highly conclusive about the multivariate analysis applied through binary logistic regressions since all show a coincidence of predictor variables and their intensity, except AM4, where the gender variable is added. The two predictor variables both with Death Anxiety and in the set of subscales are: PPE and NPPS, and, in practically all cases, their values are equal to or greater than 2, showing that social workers who have these perceptions are twice as likely to suffer the fear of their own and others’ death, both from the point of view of state and process. These data demonstrate the need to analyze the hard work performed by social workers in complex areas of their functions [51,52].

With the Fear of the Dying Process of others, it is striking that being female is a predictor variable for the risk of suffering. Different studies have shown these same findings [53,54]. This suggests that there may still be an influence of traditional roles on the caregiver’s feminized figure [55,56,57,58]. There could also be a cultural explanation because women have expressed and acknowledged their emotions in public. It has been seen as something natural, unlike men who have been forced to repress them socially and culturally [59]. Likewise, previous studies have found that women were more compassionate in their reactions to death anxiety, while men shied away from emotion and compassion [60].

This situation, which has such an impact on these professionals, demands the creation of preventive actions and programs that care for these workers’ psychological health. As we have been able to determine in other groups [11,14,42,61], there is a significant demand for psychological resources that help to cope with these extreme situations, which generate a high level of stress. Therefore, training and prevention programs should be developed that, among other consequences, reduce anxiety in the face of death. Among them, those focused on improving emotional intelligence could be of great value since they have proven helpful in different contexts [54,55,56,57,58,59,60,61,62,63,64,65].

On the other hand, it should be noted that the present study has two significant limitations. The first is that it would have been interesting to obtain a larger sample. However, it should be taken into account that the study was carried out in social and working conditions of high workload and emotional stress. On the other hand, previous studies on death anxiety in this professional group prevent us from carrying out a comparative study from a longitudinal perspective.

## 6. Conclusions

The pandemic caused by the SARS-CoV-2 coronavirus shows that many professions have been confronted with a new situation and different ways of working in record time. Although the development of their work has been possible—at least to a large extent—, the emotional variable has been of great relevance. It is one of the most important in dealing with overwhelming situations, such as those experienced by the pandemic, both personally and professionally. In this sense, controlling the inconveniences currently encountered in the face of a future scenario, either by worsening the current situation or by a new catastrophe, is essential for the best emotional and, therefore, working state.

Bearing the above in mind and considering the needs for psychological treatment in the future, workplaces must be provided with sufficient resources—material, human, and organizational—to deal with these situations. In the scenario described, these professionals have found little recognition in the institution they work in, which has aggravated the labor problem described. Therefore, it is of vital importance to value the needs provided to create a system of prevention in the face of possible new analogous situations. The question is not so much whether or not they will occur, but when they will appear.

## Figures and Tables

**Table 1:** Variables used in binary logistic regression.

**1. Gender**
Ref. Man
(1) Woman
**2. Age (Continue)**
Ref. Up to 30
31–40
41–50
51–60
>60
**3. Work**
Ref. Other
Primary Care Social Services
Specialized Social Services
Health Scope Social Services
Third Sector
**4. Whether they need psychological or psychiatric support (NPPS)**
Ref. No
Yes
**5. Whether they believe that psychological or psychiatric support should be offered from work centers (PPSS)**
Ref. No
Yes
**6. Whether they feel they may need psychological or psychiatric support (PPSN)**
Ref. No
Yes
**7. Lack of personal protection equipment (influyó en su estado de ansiedad/estrés laboral) (PPE)**
Ref. No
Yes
**8. Trabajó con la COVID-19 durante la primer ola de la pandemia (WFW)**
Ref. No
Yes
**9. Teleworked during the first wave of the pandemic (TL)**
Ref. No
Yes
**10. Recognized by the organization they work for (ROW)**
Ref. No
Yes

**Table 2:** Participants.

Title	%
**Gender**	
Woman	88.3
Man	11.7
**Age**	
Up to 30	23.0
31–40	22.4
41–50	32.6
51–60	19.7
>60	2.3
**Work**	
Primary Care Social Services	37.1
Specialized Social Services	19.9
Health social services	9.6
Third sector	24.2
Other	9.3
*n* = 304	

**Table 3:** Descriptive results.

AM1	
Yes	57.6
No	42.4
**AM2**	
Yes	59.9
No	40.1
**AM3**	
Yes	81.6
No	18.4
**AM4**	
Yes	78.3
No	21.7
**AM_TOTAL**	
Yes	68.8
No	31.3
**NPPS**	
Yes	29.2
No	70.8
**PPSS**	
Yes	86.8
No	13.2
**PPSN**	
Yes	69.1
No	30.9
**PPE**	
Yes	68.5
No	31.5
**WFW**	
Yes	48.0
No	52.0
**TL**	
Yes	81.8
No	18.2
**ROW**	
Yes	20.1
No	79.9

**Table 4:** Summary of the binary logistic regression models in MBI scales.

	B	Sig.	Exp(B)	95% CI Exp(B)	
				Lower	Superior
**AM1**					
Lack of personal protection equipment	0.994	0.000	2.701	1.548	4.714
Need psychological or psychiatric support	0.657	0.014	1.929	1.141	3.261
Constant	−1.216	0.000	0.296		
**AM2**					
Lack of personal protection equipment	0.673	0.010	1.960	1.171	3.281
Need psychological or psychiatric support	0.562	0.043	1.754	1.017	3.023
Constant	−0.673	0.341	0.805		
**AM3**					
Lack of personal protection equipment	0.772	0.018	2.163	1.145	4.088
Need psychological or psychiatric support	1.052	0.033	2.863	1.089	7.528
Constant	0.424	0.152	1.527		
**AM4**					
Lack of personal protection equipment	0.875	0.004	2.398	1.326	4.338
Need psychological or psychiatric support	1.001	0.009	2.722	1.290	5.743
Sex	0.886	0.032	2.425	1.080	5.445
Constant	−0.300	0.491	0.741		
**AM_Total**					
Lack of personal protection equipment	0.853	0.002	2.348	1.371	4.019
Need psychological or psychiatric support	0.901	0.005	2.463	1.320	4.596
Constant	−0.017	0.940	0.983		

## Data Availability

Data available on request due to privacy and ethical restrictions. Main data is contained within the article.
